# Early Sequelæ of Faucial Diphtheria and Their Treatment

**Published:** 1905-03-04

**Authors:** 


### EARLY SEQUELÆ OF FAUCIAL DIPHTHERIA AND THEIR TREATMENT.

Under the above heading Dr. G. C. Garratt [1] describes certain symptoms prone to follow a severe attack of diphtheria at the end of the first week of the illness, or in the course of the second week—at a period, that is to say, earlier than that affected by the more commonly recognised sequelæ, viz., paralyses of the palate, eyes, etc. Of these early symptoms the most prominent is vomiting, at first occasional, but later almost constant upon the act of deglutition. This vomiting, according to the author, is of nervous origin, and depends upon the act of swallowing; it is not to be confounded either with the vomiting of the acute stage, nor with that of the paralytic stage, for its treatment is not applicable to these other varieties. Approximately synchronous with the vomiting there are often apparent certain circulatory disturbances, most commonly an occasional intermission of the heart, though there may be great slowing of the pulse, even to the degree of 14 beats to the minute in the adult, or great hurrying in the heart-beats. With this there concurs some degree of cardiac dilatation, and occasionally angina and syncope. But, apart from these dramatic features, patients affected with the vomiting and cardiac intermissions above described are likely to die gradually of asthenia. Occasional symptoms are convulsions, suppression of urine, the appearance of a scarlatiniform eruption over the knees or elbows, and rapid enlargement of the liver.

The toxin of diphtheria acts very early on the grey matter of the central nervous system, and especially upon the motor nuclei, and it appears likely that the peripheral neuritis of the disease is a secondary element. Dr. Garratt also subordinates to the nervous involvement the accompanying degenerative changes to be found in the cardiac and skeletal muscles, in the kidneys, liver, spleen, lymph glands, and in the alimentary canal. The argument for the nervous basis of these lesions is as follows. Though albuminuria may be marked, there is seldom other evidence of an acute nephritis, and, if the patient survives, the disappearance of the albumin is rapid, and permanent damage to the kidney quite exceptional. Again, a patient who cannot retain a teaspoonful of water if he swallows it (and thereby excites reflexly his medullary centres) will retain 12 ounces of the same fluid given shortly after by an œsophageal tube.

As to treatment the author is of opinion that prevention can be effected by an early and adequate dose of antitoxin; if given late, that is to say after the toxin has had time to affect the grey matter, no dose of the antidote appears to affect the course of the symptoms. But assuming the existence of the condition the treatment proposed is the direct contrary of that commonly adopted. This has, of course, centred round stimulants of various descriptions. Dr. Garratt, believing the condition to indicate, in part, at all events, a hyper-æsthetic state of the medullary centres, recommends a sedative line of treatment—that is to say, the patient is to be kept absolutely recumbent, and from the first treated with large doses of bromide of sodium or potassium and of belladonna, with the administration at longish intervals, of hot water by means of an œsophageal tube. The detailed instruction for the treatment is briefly this. At the first onset of the symptoms give belladonna by the mouth, or if that be rejected, by the rectum. If vomiting continue, let nothing be swallowed, but feed by the rectum every four hours, adding tr. belladonnæ m. 20-30 to each enema. This for a child of three or four years. Pass an œsophageal (not a nasal), tube, and pour in hot water, with a little bicarbonate of soda, the water to be of a temperature of 115° Fahr. This may be at first rejected, but persevere until 10-12 ounces have been poured in. Withdraw the tube rapidly, and after a short interval put the child quietly to bed. Twice in twenty-four hours bromide of soda or potash, gr. 20, are to be added to the enemata. As the symptoms improve a gradual reduction in the doses of the drugs is to be effected, and when the child seems to be hungry, mouth-feeding is to be cautiously begun. All enemata should be given slowly, and by gravitation, and for the commencement of gastric feeding milk is less suitable than soluble albumoses or peptones, which leave no residue. Somatose with water appears to give good results.

[1] St. Bartholomew's Hospital Reports, 1904.

